# Radiation-hypersensitive cancer patients do not manifest protein expression abnormalities in components of the nonhomologous end-joining (NHEJ) pathway

**DOI:** 10.1038/sj.bjc.6600897

**Published:** 2003-04-15

**Authors:** T Leong, M Chao, S Bassal, M McKay

**Affiliations:** 1 Peter MacCallum Cancer Institute, Smorgon Family Building, St Andrews Place, East Melbourne, Victoria 3002, Australia

**Keywords:** radiation sensitivity, NHEJ, DNA double strand breaks, cancer, radiotherapy

## Abstract

Radiation therapy (RT) is utilised for the treatment of around half of all oncology patients during the course of their illness. Despite great clinical progress in the rational deployment of RT, the underlying molecular basis for its efficacy and toxicity are currently imperfectly understood. In this study, we took a biochemical approach to evaluate the potential role of key ionising radiation repair proteins in the treatment outcomes of patients with severe acute or late RT side effects. Lymphoblastoid cell lines were established from blood samples from 36 radiosensitive cases and a number of controls (the latter had had RT but did not develop significant toxicity). The expression level and migration of key proteins from the nonhomologous end-joining (NHEJ) pathway was evaluated by Western blot analysis on cases and controls. We did not observe any abnormalities in expression level or migration pattern of the following NHEJ proteins in radiosensitive cancer cases: Ku70, Ku80, XRCC4, DNA Ligase IV. These important negative results provide evidence that mutations that affect protein expression of these NHEJ components are unlikely to underlie clinical radiation sensitivity.

There is a range in the severity of normal tissue reactions observed when cancer patients receive standard radiotherapy (RT) treatment. Some individuals develop severe normal tissue reactions such as small bowel damage following pelvic irradiation or myelopathy following irradiation of the spinal cord. Normal tissue RT tolerance is dependent on many factors including dose, fractionation, the volume irradiated and individual variation in radiation sensitivity (McKay and Peters, 1997). The doses prescribed in current practice are largely based on the clinically determined tolerance of the normal tissues in the radiation field, and have evolved to limit the proportion of highly radiosensitive adverse reactions to about 0.5–5% of cases, depending on factors such as the end point under consideration (Norman *et al*, 1988). If it were possible to identify these ionising radiation (IR)-hypersensitive individuals in advance of therapy by the use of effective predictive assays, their treatment could be adjusted or changed and it might then be possible to escalate the dose in the remaining patients (i.e. the great majority of individuals). This would be expected to improve local tumour control and cure rates.

Much evidence supports a genetic basis for the predisposition to adverse normal tissue reactions after RT (McKay and Peters, 1997). Dysfunction of genes and their protein products involved in the sensing and response of cells to IR is therefore a potential mechanism of adverse normal tissue reactions to RT. Various approaches might be considered in attempts to unravel the molecular basis of clinical radiosensitivity. These include screening candidate radiosensitivity genes for mutations (Leong *et al*, 2000; Severin *et al*, 2001), adopting functional genomics approaches (e.g. using DNA microarrays) or, as adopted here, screening for abnormalities in key candidate proteins.

DNA double-strand breaks (dsbs) are the most lethal form of cellular DNA damage, and are major IR-induced DNA lesions (Jeggo, 1998a). Therefore, genes involved in processing this type of DNA lesion are excellent candidates for involvement in radiation hypersensitivity, and could account for all or a proportion of clinical RT hypersensitivity. In mammalian cells, the primary mechanism for repairing dsbs is nonhomologous end-joining (NHEJ) (Bryans *et al*, 1999). Genetic experiments indicate that the Ku heterodimer (Ku70 and Ku80), XRCC4 (X-ray repair cross complementing Chinese hamster ovary cell line) and DNA ligase IV are required for efficient NHEJ in all eukaryotic species examined so far (Jeggo, 1998a). Mutation of any one of these four factors in both the budding yeast *Saccharomyces cerevisiae* (Herrmann *et al*, 1998; Wilson *et al*, 1997) and in mammalian cells (Frank *et al*, 1998; Gao *et al*, 1998a; Grawunder *et al*, 1998) yield very similar phenotypes, the features of which include defective DNA dsb repair and marked sensitivity to IR. In mammalian cells, NHEJ also requires the catalytic subunit of DNA-dependent protein kinase (DNA-PK_CS_) (Jeggo, 1998a). However, an orthologue for this factor has not been found in the fully sequenced genome of *S. cerevisiae*. Even in species with a DNA-PK_CS_ orthologue, mutation sometimes results in a less severe phenotype than is observed for the other three components of the NHEJ pathway (Gao *et al*, 1998b; Taccioli *et al*, 1998).

Given the radiation-sensitive phenotype displayed by mutants defective in components of the NHEJ pathway, in multiple eukaryotic species, we reasoned that these proteins were good candidates for screening for abnormalities in radiation-sensitive cancer patients. An abnormality in either the abundance of protein production or its molecular mass may be because of mutations in the corresponding gene, which could then be sequenced to identify the corresponding nucleotide sequence variant. We carried out and report here the results of Western blot analysis testing for abnormalities of DNA ligase IV, XRCC4, Ku70 and Ku80 in a cohort of severely radiosensitive cancer patients.

## MATERIALS AND METHODS

### Patients (cases and controls)

**Figure 1:**
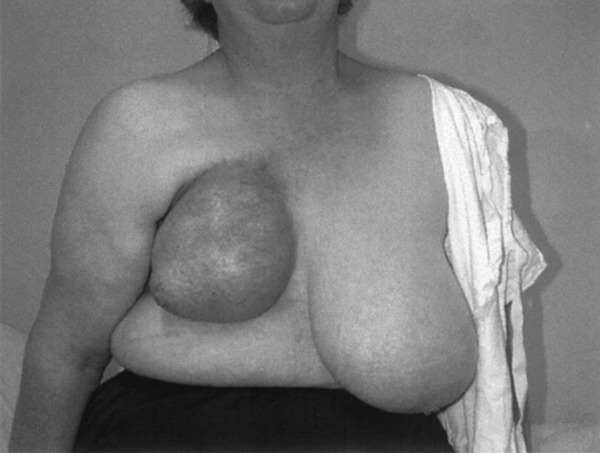
Photograph showing an example of a severe late radiation reaction. This patient (patient 8 in Table 1

The patient cohort for this study comprised 36 radiation-sensitive individuals referred by radiation oncologists at Peter MacCallum Cancer Institute and several other centres within Australia. A highly radiosensitive response was defined as a clinically overt and unexpectedly severe radiation reaction (RTOG grades 3 and 4) which occurred either acutely (during or within weeks of completion of RT) or as a late adverse reaction (months to years after completion of the RT course) (Figure 1

**Table 1:** Patient characteristics

**Patient**	**Tumour**	**Radiotherapy schedule**	**Adverse reaction**
1	Carcinoma of breast	46 Gy in 23 F plus boost 15 Gy in 5 F to breast	Severe fibrosis, retraction and telangiectasia. Rib fracture
2	Carcinoma of breast	46 Gy in 23 F plus boost 15 Gy in 5 F to breast	Severe fibrosis and retraction
3	Carcinoma of breast	50 Gy in 25 F to breast	Severe fibrosis and telangiectasia
4	Carcinoma of breast	46 Gy in 20 F to chest wall	Severe fibrosis and telangiectasia
5	Carcinoma of breast	46 Gy in 20 F to chest wall	Severe fibrosis and rib fractures
6	Carcinoma of breast	46 Gy in 20 F to chest wall	Soft tissue necrosis
7	Carcinoma of breast	50 Gy in 25 F to breast	Severe acute moist desquamation. Severe fibrosis and telangiectasia
8	Carcinoma of breast	50 Gy in 25 F plus boost 10 Gy in 5 F to breast	Severe fibrosis, retraction and telangiectasia
9	Carcinoma of breast	18 Gy in 9 F to breast (treatment terminated prematurely)	Severe acute reaction with moist desquamation, erythema and oedema
10	Carcinoma of breast	50 Gy in 25 F plus boost 10 Gy in 5 F to breast	Severe fibrosis and telangiectasia
11	Carcinoma of breast	45 Gy in 25 F plus boost 20 Gy in 10 F to breast. 48 Gy in 24 F to supraclavicular fossa and axilla	Severe fibrosis affecting breast, supraclavicular fossa and axilla, causing excessive lymphoedema
12	Carcinoma of breast (bilateral)	50 Gy in 25 F plus boost	Severe acute reaction with moist desquamation, erythema and oedema
		10 Gy in 5 F to chest wall	
13	Carcinoma of breast	50 Gy in 25 F plus boost 10 Gy in 5 F to breast	Severe acute reaction with erythema and oedema after 10 Gy
14	Carcinoma of breast	46 Gy in 23 F plus boost 15 Gy in 5 F to breast	Severe fibrosis, retraction and telangiectasia
15	Carcinoma of breast	46 Gy in 23 F plus boost 15 Gy in 5 F to breast	Severe fibrosis and retraction
16	Carcinoma of breast	45 Gy in 25 F plus boost 15 Gy in 5 F to breast	Severe acute reaction with moist desquamation and oedema. Severe fibrosis, retraction and telangiectasia
17	Carcinoma of breast	28 Gy in 14 F to breast (treatment terminated prematurely)	Severe acute reaction with moist desquamation, erythema and oedema
18	Carcinoma of tonsillar fossa	44 Gy in 22 F plus boost 16 Gy in 10 F	Severe acute moist desquamation and mucositis requiring nasogastric tube feeding for over 1 month
19	Carcinoma of cervix	45 Gy in 25 F plus 30 Gy to point A in a single insertion	Small bowel damage requiring resection. Haemorrhagic cystitis
20	Carcinoma of cervix	45 Gy in 25 F plus 30 Gy to point A in a single insertion	Strictures of sigmoid colon and ureter requiring surgery
			Peripheral neuropathy
21	Carcinoma of cervix	54 Gy in 30 F plus 25 Gy to point A in a single insertion	Acute diarrhoea and bowel obstruction requiring surgical resection
22	Carcinoma of cervix	50 Gy in 25 F plus 25 Gy to point A in a single insertion	Severe small bowel damage
23	Carcinoma of anus	28 Gy in 14 F (treatment terminated prematurely)	Severe moist desquamation
24	Malignant melanoma	50 Gy in 25 F to supraclavicular fossa	Severe moist desquamation during 4th week of RT
25	Carcinoma of floor of mouth	58 Gy in 29 F (treatment terminated prematurely)	Severe acute mucositis
			Late radionecrosis of mandible requiring resection
26	Malignant thymoma	50 Gy in 25 F to chest	Severe and prolonged acute oesophagitis for 6 weeks
27	Carcinoma of lung	60 Gy in 30 F to chest	Subacute pneumonitis
28	Carcinoma of lung	36 Gy in 12 F	Acute, marked confluent erythema of skin. Subacute pneumonitis
29	Transitional cell carcinoma of urethra	50 Gy in 25 F to pelvis	Avascular necrosis of hip
30	Carcinoma of prostate	64 Gy in 32 F to pelvis	Radiation proctitis with PR bleeding requiring laser treatment
31	Carcinoma of prostate	60 Gy in 30 F to pelvis	Radiation proctitis with severe PR bleeding
32	Carcinoma of prostate	66 Gy in 33 F to pelvis	Radiation proctitis with severe PR bleeding
33	Carcinoma of prostate	64 Gy in 32 F to pelvis	Radiation proctitis with severe PR bleeding
34	Carcinoma of prostate	66 Gy in 33 F to pelvis	Radiation proctitis with PR bleeding requiring laser treatment
35	Carcinoma of prostate	64 Gy in 32 F to pelvis	Radiation proctitis with PR bleeding requiring laser treatment
36	Carcinoma of prostate	64 Gy in 32 F to pelvis	Radiation proctitis with severe PR bleeding

Gy=gray; F=fractions.

) developed severe fibrosis, retraction and telangiectasia of the breast following routine postoperative RT for breast cancer.

). A number of cases had controls (nonradiosensitive cancer patients) which were matched for the following factors: sex, age, tumour type, stage and grade and medications. The study was approved by the Peter MacCallum Cancer Institute Ethics Committee and all patients gave informed consent.

### Lymphoblastoid cell lines

Blood was collected from each patient, from which lymphocytes were isolated by Ficoll gradient centrifugation and then transfected with Epstein–Barr virus to produce B-lymphoblastoid cell lines (LCLs) (Neitzel, 1986). These transformed lymphocytes were propagated as described (Leong *et al*, 2000) to provide an accessible source of protein extracts.

### SDS–polyacrylamide gel electrophoresis and Western blotting analysis

Protein extracts were prepared from whole patient LCLs by sonication in SDS sample buffer. Equal amounts of protein from cases and controls (50 *μ*g lane^−1^ for ligase IV and XRCC4 gels; 15 *μ*g lane^−1^ for Ku70 gels; 5 *μ*g lane^−1^ for Ku80 gels) were loaded onto a 10% SDS–PAGE gel and subjected to electrophoresis. For Western blot analysis, separated proteins were transblotted onto PVDF membrane (PolyScreen PVDF Transfer Membrane, NEN Life Science Products, Boston, MA, USA) at a constant voltage of 100 V for 2 h in transfer buffer (25 mM Tris, 190 mM glycine, 20% v v^−1^ methanol), and the membrane blocked overnight in 10% skim milk. The membrane was probed with anti-DNA ligase IV (at 1000-fold dilution), anti-XRCC4 (at 2500-fold dilution), anti-Ku70 (at 15 000-fold dilution) or anti-Ku80 (at 15 000-fold dilution) antibodies as the primary antibody and horseradish peroxidase conjugated goat anti-rabbit IgG as the secondary antibody (BIO-RAD, CA, USA). Protein products were then visualised by chemiluminescence (NEN Life Science Products, Boston, MA, USA) and autoradiography. We used gamma tubulin antibody (Wise *et al*, 2000) as an internal control to adjust for differences in the amount of protein loaded in each lane. The anti-DNA ligase IV antibody was raised against the C-terminal portion of the protein, while antibodies to XRCC4, Ku70 and Ku80 were raised against the full length of the protein. All primary antibodies were kindly provided by Drs Susan Critchlow and Stephen Jackson (Wellcome/CRC Institute, Cambridge, UK).

## RESULTS

**Figure 2:**
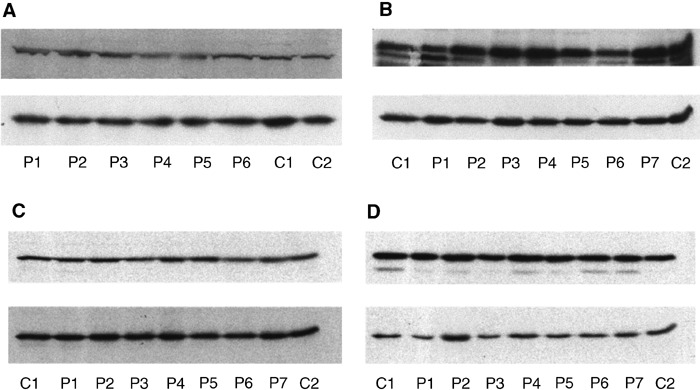
Typical Western blot analysis of protein extracts from radiosensitive patients using (**A**) anti-DNA ligase IV, (**B**) anti-XRCC4, (**C**) anti-Ku70 and (**D**) anti-Ku80 antibodies. There were no differences in expression for any of the four proteins between radiosensitive and control patients. Gamma-tubulin was used as an internal control to adjust for differences in the amount of protein loaded in each lane (lower autoradiograph in each panel). P=radiosensitive patient, C=control patient.

A total of 36 patients were examined for evidence of defects in DNA ligase IV, XRCC4, Ku70 and Ku80 proteins using Western blotting (Table 1). Bands of reported and appropriate size were observed in all cases. Apparent molecular masses for DNA ligase IV, XRCC4, Ku70 and Ku80 proteins were 96, 55, 70 and 80 kDa, respectively. The screening analysis revealed no evidence of differences in expression level or of abnormal protein forms for any of the four proteins in the 36 radiosensitive patients tested, as compared to control patients (Figure 2

). We would not expect any variation in protein levels for these NHEJ proteins in control patients.

## DISCUSSION

In this study, we adopted a candidate protein approach to ascertain whether abnormalities in proteins which collaborate in the major dsb repair pathway, NHEJ, could be detected in cancer patients who had suffered severe RT side effects in their normal tissues. We therefore set out to determine whether defects in DNA ligase IV, XRCC4, Ku70 or Ku80 could account at least for some cases of radiation hypersensitivity. The screening analysis was performed using Western blotting. This method should detect any obvious differences in protein expression between radiosensitive and control patients which may have resulted from mutations in the corresponding genes. Despite an extensive analysis, we did not detect any differences in expression in the cohort of patients tested. We had also attempted to examine DNA-PK_CS_ using Western blotting. However, because of the large molecular mass of DNA-PK_CS_ (460 kDa), initial attempts proved unsuccessful. Despite efforts to optimize the procedure by varying the parameters for electrophoresis and transblotting, we were unable to successfully perform the Western analysis for DNA-PK_CS_.

Although we failed to identify any NHEJ protein defects, this research is important for a number of reasons. First, studies such as this have the potential to contribute to the understanding of the mechanistic basis for clinical radiation hypersensitivity, which is currently largely unknown. Second, numerous mammalian cell lines and animals with naturally occurring or genetically engineered mutations in NHEJ components manifest radiosensitivity, including in the heterozygous state (see Introduction). The latter might be expected to be most likely to be revealed in screening studies such as reported here. Third, there is a precedent for detection of abnormal ligase IV on Western analysis in a radiosensitive individual: a leukaemia patient with fatal radiosensitivity has been identified to carry a mutation in a NHEJ protein, with an abnormal pattern on Western analysis (Riballo *et al*, 1999). Although this patient appears to have suffered a more dramatic adverse radiation reaction compared to the patients in our cohort, it is difficult to make direct comparisons. There is currently no clear picture of the spectrum of severe adverse radiation reactions that are seen in the clinical setting. One factor determining the outcome of an adverse radiation reaction is the organ that has been irradiated. For example, the consequences of a severe reaction in skin (e.g. fibrosis) would be far less dramatic than that seen in the spinal cord (e.g. paraplegia). This patient with a mutation in ligase IV was a young child who had received radiation to a developing brain. There are no similar patients in our cohort who had received cranial irradiation for comparison. We aimed to further evaluate this reported experience to determine its generalisability to other radiosensitive cancer patients. Collectively, these considerations formed a logical rationale for the present investigations.

Although a variety of factors could theoretically influence radiosensitivity, strong evidence implicates a genetic basis for the predisposition to adverse normal tissue RT reactions. In recent years, realisation of a probable genetic basis for radiosensitivity has led to searches for genetic aberrations that could be used as predictive assays. Screening for heterozygous mutations in the gene homozygously mutated in the cancer-prone, radiosensitivity disorder, ataxia-telangiectasia (*ATM*: AT mutated) in radiosensitive patients has been vigorously pursued during the past few years. However, data from several studies suggest that ATM gene defects are not a major cause of radiation hypersensitivity (Appleby *et al*, 1997; Clarke *et al*, 1998; Hall *et al*, 1998; Ramsay *et al*, 1998; Shayeghi *et al*, 1998; Oppitz *et al*, 1999). We have previously reported the results of mutation analysis of BRCA1 and BRCA2 cancer predisposition genes in a cohort of radiation-hypersensitive cancer patients (Leong *et al*, 2000).

Of the many types of DNA damage that can occur, dsbs are a critical event leading to cell death if the break remains unrepaired or is repaired improperly. Studies with mammalian cells have suggested that the majority of dsbs are rejoined by NHEJ. Studies using IR-sensitive mutant rodent cells have identified five factors involved in this process; Ku70, Ku80, XRCC4, DNA ligase IV and DNA-PK_CS_. Further investigation of these mutants revealed that these proteins were also required for V(D)J recombination, the process in B and T cells which allows immunological diversity during antibody and T-cell receptor production, indicating that these two fundamental DNA transactions share common components (Pergola *et al*, 1993).

Several murine and human models have highlighted the critical role that all these factors play in DNA dsb repair. Targeted disruption of the gene encoding DNA ligase IV leads to late embryonic lethality and impaired V(D)J recombination in mice (Barnes *et al*, 1998; Frank *et al*, 1998). Ligase IV-deficient embryonic fibroblasts also show marked sensitivity to IR (Frank *et al*, 1998). 180BR is a radiosensitive fibroblast cell line derived from a leukaemia patient who over-responded to RT and subsequently died of radiation morbidity. 180BR cells were shown to harbour a homozygous missense mutation in the ATP binding domain of human DNA ligase IV (Riballo *et al*, 1999). DNA ligase IV mutations have also been identified recently in several patients exhibiting developmental delay and immunodeficiency, and cell lines derived from these patients exhibit pronounced radiosensitivity (O'Driscoll *et al*, 2001). XRCC4 forms a tight complex with DNA ligase IV in mammalian cells. Recombinant XRCC4 protein stimulates DNA binding and the ligation activity of DNA ligase IV *in vitro* (Grawunder *et al*, 1997). Mice with targeted disruption of the gene encoding XRCC4 have growth defects, premature senescence, impaired V(D)J recombination and marked sensitivity to IR (Gao *et al*, 1998a). Ku has at least three separate functions in end-joining DNA dsb repair that have been identified *in vitro*. It generally facilitates end-joining by aligning DNA ends, and it specifically recruits both XRCC4-ligase IV and DNA-PK_CS_ to DNA ends (Nick_McElhinny *et al*, 2000). Ku80-deficient ES cells and pre-B-cell lines are hypersensitive to IR (Nussenzweig *et al*, 1997) and consistent with the radiation-hypersensitive phenotype of the cell lines, Ku80 mutant mice also display extreme radiosensitivity (Nussenzweig *et al*, 1997). Mice lacking Ku70 are immunodeficient and growth retarded, and Ku70-deficient ES cells have increased radiosensitivity, defective DNA end binding activity and an inability to support V(D)J recombination (Gu *et al*, 1997a, 1997b). In mammalian cells, NHEJ also typically requires DNA-PK_CS_. DNA-PK_CS_ is a member of the phosphatidylinositol (PI) 3-kinase family that is activated upon binding to DNA ends. Cells derived from highly radiosensitive SCID mice have a DNA dsb repair deficiency caused by a mutation in the DNA-PK_CS_ gene. However, there are circumstances where mutation of DNA-PK_CS_ still allows much greater levels of end-joining than are observed when Ku, XRCC4 or ligase IV is mutated. For example, mice completely deficient in DNA-PK_CS_ can join signal end intermediates in V(D)J recombination, and ES cells from such mice possess a normal level of resistance to IR (Gao *et al*, 1998b; Taccioli *et al*, 1998). The function of DNA-PK_CS_ in NHEJ therefore may be more dispensable than that of Ku, XRCC4 or ligase IV, depending on the organism, cell type and molecular context of the ends to be joined (Nick_McElhinny *et al*, 2000).

In summary, the involvement of DNA ligase IV, XRCC4, Ku70 and Ku80 in dsb repair, and the radiosensitive phenotype displayed by mouse and human mutants in these NHEJ components, justified analysis of these proteins in our cohort of radiation-hypersensitive patients. We screened a highly selected group of cancer patients with severe adverse reactions to standard RT for defects in four of the five major NHEJ components using Western analysis: no defects were detected. These results suggest that mutations that affect protein expression of these factors do not account for most cases of clinical radiation hypersensitivity, and that screening for abnormalities of these factors using Western blotting might be unlikely to be useful for predicting clinical response to RT. However, we have not completely excluded that defects in the NHEJ pathway may contribute to clinical radiosensitivity. It is possible that mutational changes that confer radiosensitivity but have no other easily detectable impact may be missense or subtle mutations that may not affect dramatically protein levels. Also, we have not excluded that defects in DNA-PK_CS_ might contribute to clinical radiosensitivity. Since mutations of DNA ligase IV account for some instances of radiation hypersensitivity, we are examining further radiosensitive individuals for abnormal DNA ligase IV protein expression. Ongoing candidate gene/protein analyses in radiosensitive cancer patients are expected to yield further examples of the range of molecular defects causing human radiosensitivity.
